# Altering Moisture Transport Mechanisms in Multilayer Material Systems via Open Channel Networks for Enhanced Comfort

**DOI:** 10.1021/acsomega.6c07464

**Published:** 2026-10-12

**Authors:** Ada Gulbinienė, Vaida Dobilaitė, Milda Jucienė

**Affiliations:** † Faculty of Mechanical Engineering and Design, 70309 Kaunas University of Technology, Studentu Street 56, Kaunas LT-44029, Lithuania; ‡ Institute of Architecture and Construction, Kaunas University of Technology, Tunelio Street 60, Kaunas LT-44029, Lithuania

## Abstract

This study examines how the composition and structural configuration of two- and three-layer polymer-based systems influence thermophysiological comfort by altering water vapor transport mechanisms. Open moisture-transport channel networks were introduced into the top layer via controlled perforation to modify the dominant vapor transmission pathways. The results reveal nonadditive sorption behavior in multilayer systems, in which low-permeability outer layers constrain overall moisture transport. The incorporation of engineered open channels mitigates this limitation by enabling an additional Fickian diffusion pathway, thereby enhancing water vapor permeability and moisture uptake. The effect is particularly pronounced in systems containing a synthetic leather layer. Increasing the moisture transport channel coefficient to 0.0145 enhanced the water vapor permeability of synthetic leather by up to 3.5 times and of natural leather by up to 1.5 times. Consequently, the water vapor absorption of the corresponding multilayer systems increased by 14–73 times (from 0.03–0.60 to 2.26–8.33 mg cm^–2^) for synthetic leather systems and by 45–61% (from 12.8–20.1 to 18.6–32.3 mg cm^–2^) for natural leather systems. This work establishes a relationship between layer-specific properties and system-level moisture transport, demonstrating that targeted modification of vapor transfer pathways can effectively enhance thermophysiological comfort and moisture management in multilayer material systems, particularly in clothing and accessory applications.

## Graphical abstract

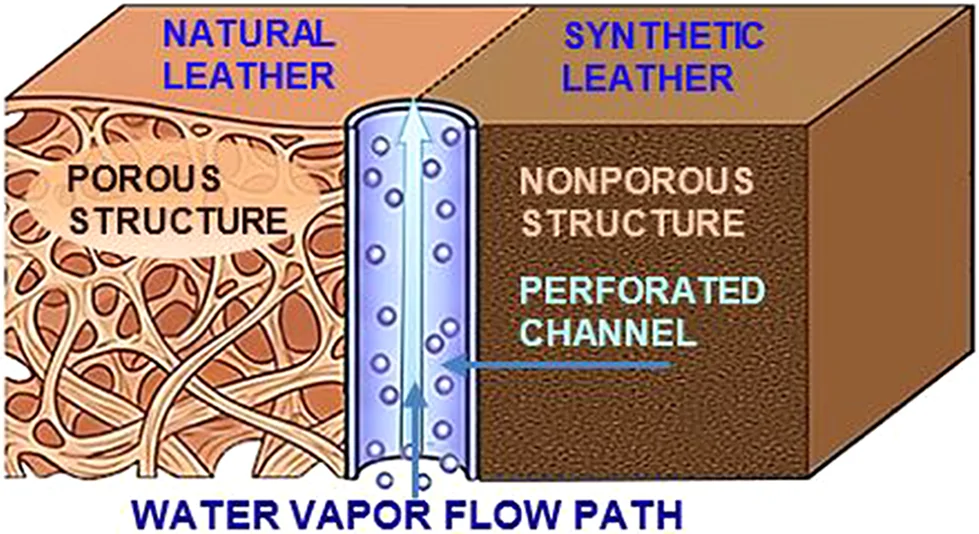

## 1 Introduction

Multilayer polymer-based material systems are frequently used in clothing and accessory applications, where their performance is governed by structure–property relationships that control heat and mass transfer. In such systems, thermophysiological comfort is primarily determined by the coupled mechanisms of water vapor transport and moisture sorption across individual layers. Each component contributes distinctly to overall transport behavior, and the resulting performance cannot be described as a simple superposition of individual layer properties.^1,2^

Moisture management, in particular, represents a critical functional requirement in footwear, where inadequate vapor transport can lead to discomfort, reduced thermal efficiency, and compromised hygienic conditions.^3–9^ The human foot exhibits one of the highest densities of sweat glands in the body, especially on the plantar surface, necessitating efficient regulation of the local microclimate.^10^ In footwear systems, moisture is partially absorbed by the upper and subsequently transported through the multilayer assembly, while a substantial fraction is retained within the insole. Under intensive conditions, insole materials can absorb the majority of the generated moisture, highlighting their central role in moisture buffering and redistribution.^11,12^ Consequently, the selection and structural integration of upper, lining, and insole materials are critical for achieving effective moisture management.

Material design strategies aimed at improving comfort performance have focused on both the composition and architecture. Polymer-based insole systems incorporating materials such as polyethylene, polypropylene, ethylene vinyl acetate, and polyurethane have demonstrated improved functional properties.^13^ Similarly, multilayer assemblies incorporating natural fibers, including bamboo-based textiles, have shown enhanced hygienic and mechanical performance.^14^ However, increasing structural complexity often leads to reduced water vapor permeability, indicating the presence of interfacial transport resistance and nonadditive behavior in multilayer systems.^15^

Advanced processing approaches, particularly laser-based techniques, enable precise control over material microstructure and associated transport properties.^16–21^ Laser perforation allows the formation of well-defined pore architectures and channel networks that can facilitate moisture transport. For example, engineered textile systems with integrated transport channels have demonstrated improved sweat wicking, evaporation, and cooling performance.^17^ It has been determined that the microporous structures have been shown to promote directional liquid transport and efficient moisture removal from the skin.^16,22–24^ The spatial distribution and geometry of such channels play a key role in governing coupled heat and mass transfer in layered systems.^25^

Despite these advances, a mechanistic understanding of how structural modifications alter water vapor transport pathways in multilayer polymer systems remains limited. In particular, the role of engineered open-channel networks in overcoming transport limitations imposed by low-permeability layers has not been systematically addressed. Therefore, this study investigates two- and three-layer footwear material systems in which open moisture transport channels are introduced into the lining layer to modify the dominant vapor transfer mechanisms and to optimize moisture management and thermophysiological comfort in advanced multilayer polymer systems.

## 2 Materials and Methods

### 2.1 Materials

The materials selected for this study are representative of the internal footwear insole systems used in commercial footwear, including individual components and their two- and three-layer systems. Natural and synthetic leathers were used as the lining layer, natural cork as the intermediate layer, and cellulose-based cardboard as the bottom insole layer (Table 1.).

**Table 1:** Characteristics of the Materials

notation	materials	thickness, mm	density, mg mm^–3^	*P*, mg cm^–2^ h^–1^
NL	natural chrome-tanned lining leather (*JSC Naturali oda & Ko*, Lithuania)	2.635	0.431	2.04
SL	synthetic leather (PVC Synthetic Roll) (*Duletai*, China)	0.97	0.445	0.45
NC	natural cork (*The Felt Store*, Canada)	2.5	0.231	2.82
CF	cardboard FINTEX 50 (*Holtex*, Poland)	2.25	0.566	2.26

During the preparation of the multilayer material systems and throughout the testing, the individual layers were mechanically stacked without adhesive. This configuration was intentionally selected to evaluate the intrinsic moisture transport properties of the material systems without the additional influence of adhesive layers. The selected materials enable systematic evaluation of structure–property relationships governing moisture transport in multilayer configurations. Material characteristics are presented in Table 1.

The top layer of the material systems was perforated by mechanical cutting to introduce open cylindrical through-thickness channels aimed at enhancing moisture transport and overall comfort performance in two- and three-layer systems. The material specimens were mechanically perforated by a specialized company (Fantas, Lithuania) using a CAD/CAM CNC leather punching machine. The channel geometry was defined by the diameters of *d*_1_ = 0.7 ± 0.1 mm and *d*_2_ = 1.0 ± 0.1 mm, while the channel densities were *ρ*_1_ = 0.71 cm^–2^, *ρ*_2_ = 1.27 cm^–2^, and *ρ*_3_ = 1.84 cm^–2^. The corresponding geometrical parameters of the channel networks are presented in Table 2.

**Table 2:** Parameters of the Moisture Transfer Channels Coefficient *k*_ch_ × 10^–2^

*d*_*i*_, mm		
ρ_j_, cm^–2^	0.7	1.0
0	0	0
0.71	0.27	0.56
1.27	0.49	0.99
1.84	0.71	1.45

To quantify the structural modification, a moisture transport channel coefficient was introduced, representing the fraction of the surface area occupied by the channels

1 $$k_{ch}=S_{d_{i}}\cdot \rho_{j}$$

where *S* *_di_* is the area of the open channel, cm^2^; *ρ*_*j*_ is the moisture transfer channel density, cm^–2^.

### 2.2 Mass, Thickness, and Apparent Density of the Test Pieces

The mass, thickness, and apparent density of the investigated specimens were determined in accordance with ISO 2419:2004. For each material, three square specimens (100 × 100 mm) were prepared under controlled thickness and mass conditions. Specimen mass was measured using an AB 104-S analytical balance (*Mettler Toledo*, Switzerland) with an accuracy of 0.1 mg. Thickness measurements were performed following ISO 2589:2016 using a UI-FT43 thickness gauge under a contact pressure of 49 ± 5 kPa. Reported values represent the mean of three independently prepared specimens. The confidence level and coefficient of variation for each material group were within 3–5%.

The apparent densities of the specimens were determined according to ISO 2420:2002. The density was calculated as the ratio of mass to volume, where the volume was obtained from the measured thickness and specimen area. The reported values correspond to the average of three measurements for each material with coefficients of variation not exceeding 4%.

### 2.3 Comfort Property Measurement

Before testing, all specimens of individual layers and multilayer systems were conditioned under standard atmospheric conditions in accordance with LST EN 12222:1997.

Water vapor permeability was determined according to LST EN ISO 14268 under controlled temperature and relative humidity conditions. Each specimen was placed over a test container filled with a dry solid silica gel desiccant. The layers of the multilayer system specimens were mechanically stacked without adhesive. The assembled setup was maintained in a conditioned environment at a temperature of 20 ± 2 °C and a relative humidity of 65 ± 5%. The prepared containers were placed in the holder of an STM 473 device (*SATRA*, UK) and tested under dynamic conditions throughout the measurement period. Water vapor permeability was calculated using eq 2.

2 $$P_{WV}=\frac{M_{2}-M_{1}}{S_{1}\cdot t}$$

where *P*_WV_ is the water vapor permeability, mg cm^–2^ h^–1^; *M*_1_ represents the initial mass of the desiccant, mg; *M*_2_ is the desiccant mass after the test, mg; *S*_1_ is the surface area of the sample, cm^2^; *t* is the duration of the test, h.

Water vapor absorption was determined in accordance with LST EN ISO 17229. In this method, the test specimen (or the layers of the multilayer system specimen, mechanically stacked without an adhesive) was clamped together with an impermeable reference material over the opening of a container containing 50 mL of water. The prepared containers were placed in an STM 478 device (*SATRA*, UK) and maintained under controlled conditions throughout the test period. Water vapor absorption was calculated based on the difference in specimen mass before and after the test by eq 3.

3 $$A_{WV}=\frac{M_{2}-M_{1}}{S_{2}}$$

where *A*_WV_ is the water vapor absorption, mg cm^–2^; *M*_1_ is the initial mass of the sample, mg; *M*_2_ is the mass of sample after test, mg; *S*_2_ is the sample surface area, cm^2^.

## 3 Results and Discussion

### 3.1 Comfort Properties of Multilayer Systems

The plantar surface of the human foot is in direct contact with the lining of the shoe-insole system. Sweat released from the foot is first absorbed by the lining material and subsequently diffused into the deeper layers of the system. To investigate how the comfort properties of shoe insole systems depend on their composition, the water vapor permeability and absorption of individual materials (NL, SL, NC, CF) and their corresponding two- and three-layer systems were evaluated. Natural (hydrophilic) and synthetic (hydrophobic) leathers with differing structural and physicochemical properties were used as the top-layer materials. The results of water vapor absorption for individual materials and their multilayer systems are presented in Figures 1 and 2.

**Figure 1:**
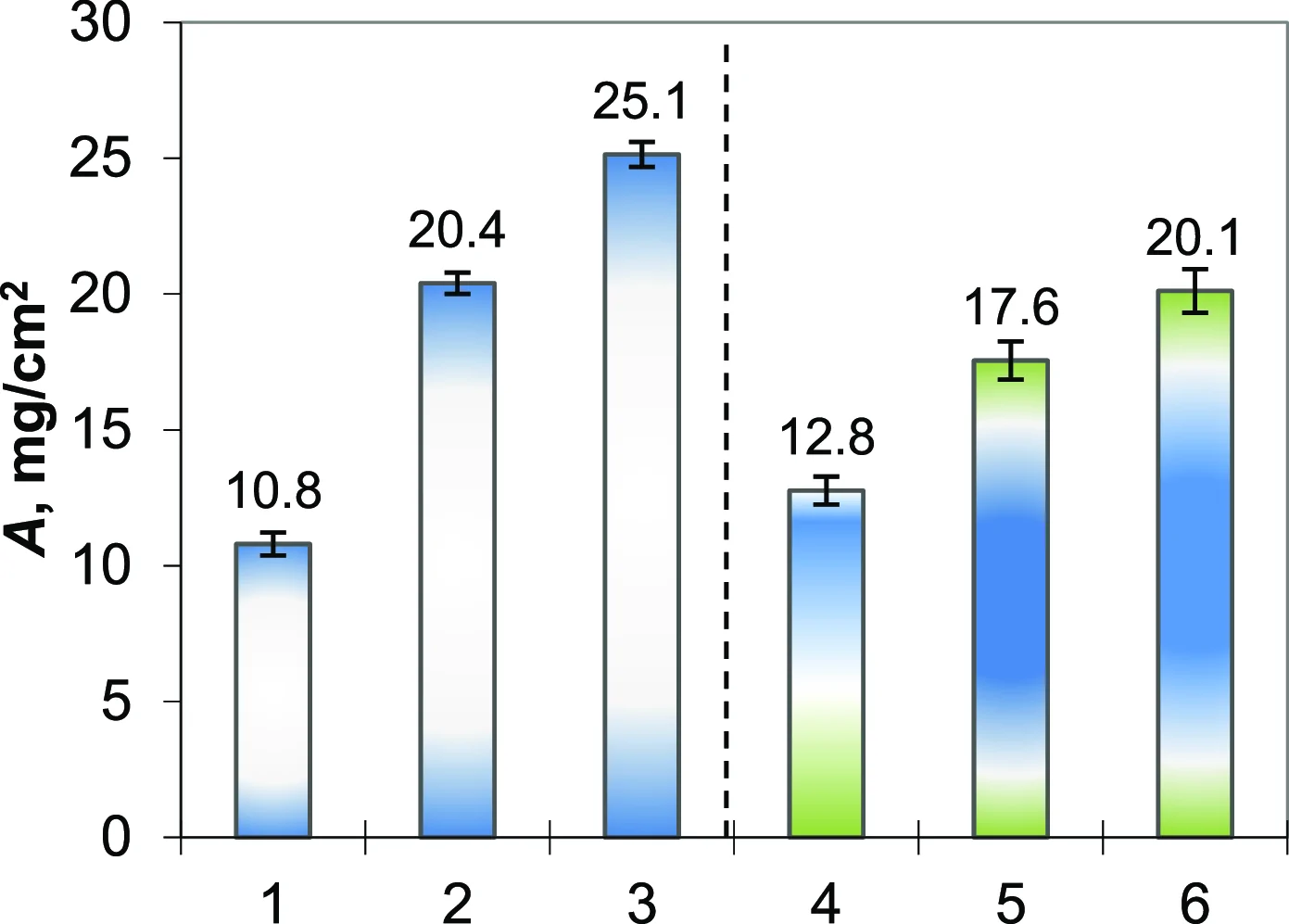
Water vapor absorption of materials (1 – NL; 2 – NC; 3 – CF) and their systems (4 – NL + NC; 5 – NL + CF; 6 – NL + NC + CF).

**Figure 2:**
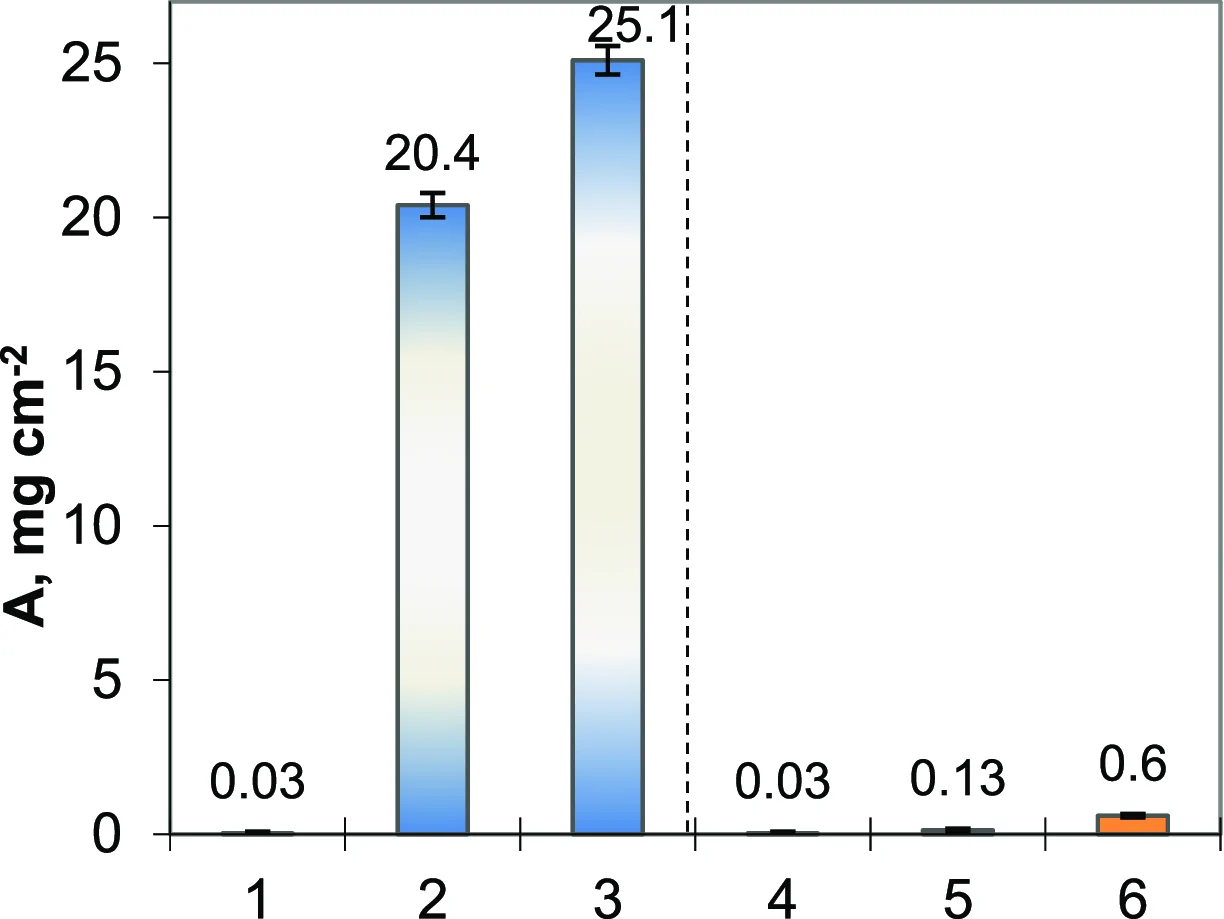
Water vapor absorption of materials (1 – SL, *SD* = 0.031; 2 – NC; 3 – CF) and their systems (4 – SL + NC, *SD* = 0.048; 5 – SL + CF, *SD* = 0.050; 6 – SL + NC + CF, *SD* = 0.048).

The water vapor transport driving force through the multilayer material systems is the vapor concentration gradient. The moisture sorption behavior of multilayer material systems depends on the water vapor absorption capacity of individual layers and their water vapor permeability. Each component contributes differently to overall moisture transport.^28–30^ The water vapor permeability results for the single layers are presented in Table 1. As shown, all single layers except synthetic leather exhibit high water vapor permeability (2.04–2.82 mg cm^–2^ h^–1^). These values are 4.5–6.2 times higher than those of synthetic leather (0.45 mg cm^–2^ h^–1^).

Similarly, all single layers except synthetic leather demonstrate high water vapor absorption (10.8–25 mg cm^–2^). The water vapor absorption of natural cork and cardboard is up to two times higher than that of natural leather. In contrast, synthetic leather exhibits negligible water vapor absorption (0.03 mg cm^–2^).

The results reveal the nonadditive sorption behavior in multilayer systems. The sorption capacity of multilayer systems is lower than the combined capacity of their constituent layers, particularly when the top layer exhibits low water vapor permeability. Water vapor absorption also depends on the number of constituent layers (Figures 1 and 2). The three-layer system (NL + NC + CF) exhibits approximately 1.2 and 1.6 times higher absorption than the corresponding two-layer systems (NL + NC and NL + CF). Although the individual layers show relatively high sorption capacity, the assembled systems (NL + NC, NL + CF, and NL + NC + CF) absorb only 50%, 41%, and 36% of the total sorption capacity of their constituent materials, respectively (Figure 1).

In contrast, all two- and three-layer systems with a synthetic leather top layer exhibit negligible sorption behavior (Figure 2). Their water vapor absorption values are nearly identical to those of synthetic leather, with the systems (SL + NC, SL + CF, and SL + NC + CF) absorbing only 0.15%, 0.5%, and 1.3% of their total layers’ sorption capacity, respectively. Due to its low water vapor permeability, synthetic leather acts as a barrier, restricting moisture transport to the inner layers of the system. Consequently, the water vapor absorption of two-layer systems with a natural leather top layer (NL + NC and NL + CF) is approximately 425 and 135 times higher than that of the corresponding systems with synthetic leather (SL + NC and SL + CF). Similarly, the three-layer system (NL + NC + CF) exhibits approximately 34 times higher absorption than the corresponding SL + NC + CF system.

The nonadditive sorption behavior observed in the multilayer systems may be attributed to several factors. The top layer, particularly synthetic leather (SL) and, to a lesser extent, natural leather (NL), exhibits lower water vapor permeability than the inner layers (NC and CF). Consequently, the top layer acts as a barrier, restricting the transport of water vapor to the underlying layers. In addition, NL possesses a relatively high water vapor sorption capacity, causing a portion of the incoming water vapor to be retained within its collagen structure before reaching the lower layers. As a result, the water vapor concentration gradient across the inner layers is reduced, thereby decreasing the driving force for moisture transport and sorption in the subsequent layers. Consequently, the total water vapor absorption of the multilayer system is lower than the sum of the absorption capacities of its individual component layers. This behavior is governed by the water vapor transport characteristics of the top layer and likely contributes to the observed nonadditive sorption behavior.

The differing nature and properties of the NL and SL layers significantly influence the mechanism of water vapor transport. Figure 3 illustrates the water vapor transport mechanisms in natural (NL) and synthetic (SL) leather. Natural leather (NL) possesses a hierarchically porous collagen-fiber structure with interconnected pores and capillaries across a broad size distribution.^31,32^ This architecture promotes efficient water vapor transport under a vapor concentration gradient through coupled diffusion pathways. In larger pores, transport is governed predominantly by Fickian diffusion, whereas in pores approaching the molecular mean free path of water vapor, wall–molecule interactions become dominant, leading to Knudsen diffusion.^33,34^ Consequently, water vapor transport in NL is governed by a combined Fickian–Knudsen diffusion mechanism through the interconnected porous network (Figure 3a), which accounts for the intrinsically high vapor permeability of natural leather. Under these conditions, the dominant water vapor transfer flux *J*_FK_ is governed by Fick’s law (eq 4).

$$J_{FK}=-D_{eff}\frac{dC}{dx}$$ 4

where *J*_FK_ is the water vapor transfer flux through the interconnected porous network, *dC*/*d* *x* is the vapor concentration gradient, and *D*_eff_ is the effective diffusion coefficient in porous NL.

**Figure 3:**
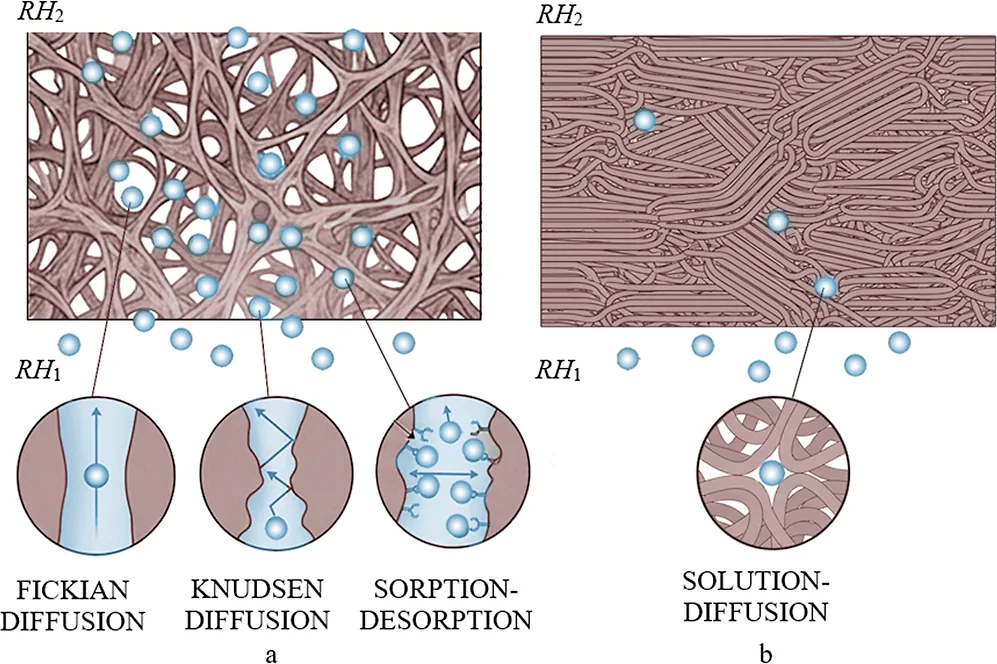
Water vapor transfer mechanisms in natural leather (a) and synthetic leather (b); *RH*_1_ > *RH*_2_.

As the pore diameter approaches the mean free path of water vapor molecules, molecule–wall interactions become dominant over intermolecular collisions, and vapor transport proceeds via Knudsen diffusion. Under these conditions, the effective diffusion coefficient can be expressed as

5 $$\frac{1}{D_{eff}}=\frac{\tau }{\varepsilon }\left(\frac{1}{D_{molecular}}+\frac{1}{D_{Knudsen}}\right)$$

where *ε* is the porosity factor and *τ* is the tortuosity factor in porous NL.

In the microporous, hydrophilic NL layer, which contains active peptide and ionized groups, water molecules not only diffuse through pores, capillaries, and intermolecular spaces but also interact with polar functional groups within the collagen matrix. These interactions promote adsorption onto the leather surface, followed by desorption into the interfacial region, giving rise to an additional sorption–desorption transport pathway (Figure 3a) associated with the water vapor transfer flux (*J*_sorp/desorp_).^32,33^ The high water vapor absorption capacity of NL is therefore attributed to strong interactions between water molecules and peptide and ionized groups, which also contribute to its overall vapor permeability.

Water vapor transport through natural leather (NL) proceeds via a dual mechanism comprising combined Fickian–Knudsen diffusion within the porous network and sorption–desorption transport through the collagen matrix (Figure 3a)

6 $$J_{NL}=J_{FK}+J_{sorp/desorp}$$

The water vapor transfer through nonporous materials is governed by the available free volume within the polymer matrix. A higher free volume results in a higher sorption capacity and molecular mobility and is closely related to the polymer’s density and physical state.^34–36^ In such systems, vapor transport proceeds via a solution–diffusion mechanism. In the nonporous hydrophobic surface layer structure of the synthetic leather (SL), water molecules migrate between rigid, closely packed polymer chains (Figure 3b), occupying free-volume sites and moving between them. The water molecules dissolve into and diffuse through the polymer free volume by the water vapor transfer flux (*J*_sol‑diff_). Due to its hydrophobic nature and nonporous morphology, SL exhibits negligible moisture absorption and limited water vapor permeability.

The differences in the structure of the NL and SL layers and their water vapor transport mechanisms significantly influence the moisture transport behavior and comfort properties of multilayer systems. Therefore, introducing open channel networks to modify the water vapor transport mechanism and reduce the barrier effect of the top layer provides an effective approach to controlling mass transfer and improving the comfort properties of multilayer systems.

### 3.2 Modification of Water Vapor Transfer Mechanisms and Influence on Comfort Properties

The sorption capacity of multilayer systems is lower than the combined capacity of their constituent layers, particularly when the top layer exhibits low water vapor permeability. To address this limitation, the top layer was perforated to form a network of open cylindrical through-thickness moisture transport channels (NL, SL), leading to a pronounced increase in water vapor permeability (Figure 4). Perforation induces distinct transport responses depending on the material type: natural leather exhibits a linear dependence on the square root of the moisture transport channel coefficient (*k*_ch_^0.5^), whereas synthetic leather exhibits an approximately linear dependence on *k*_ch_.

**Figure 4:**
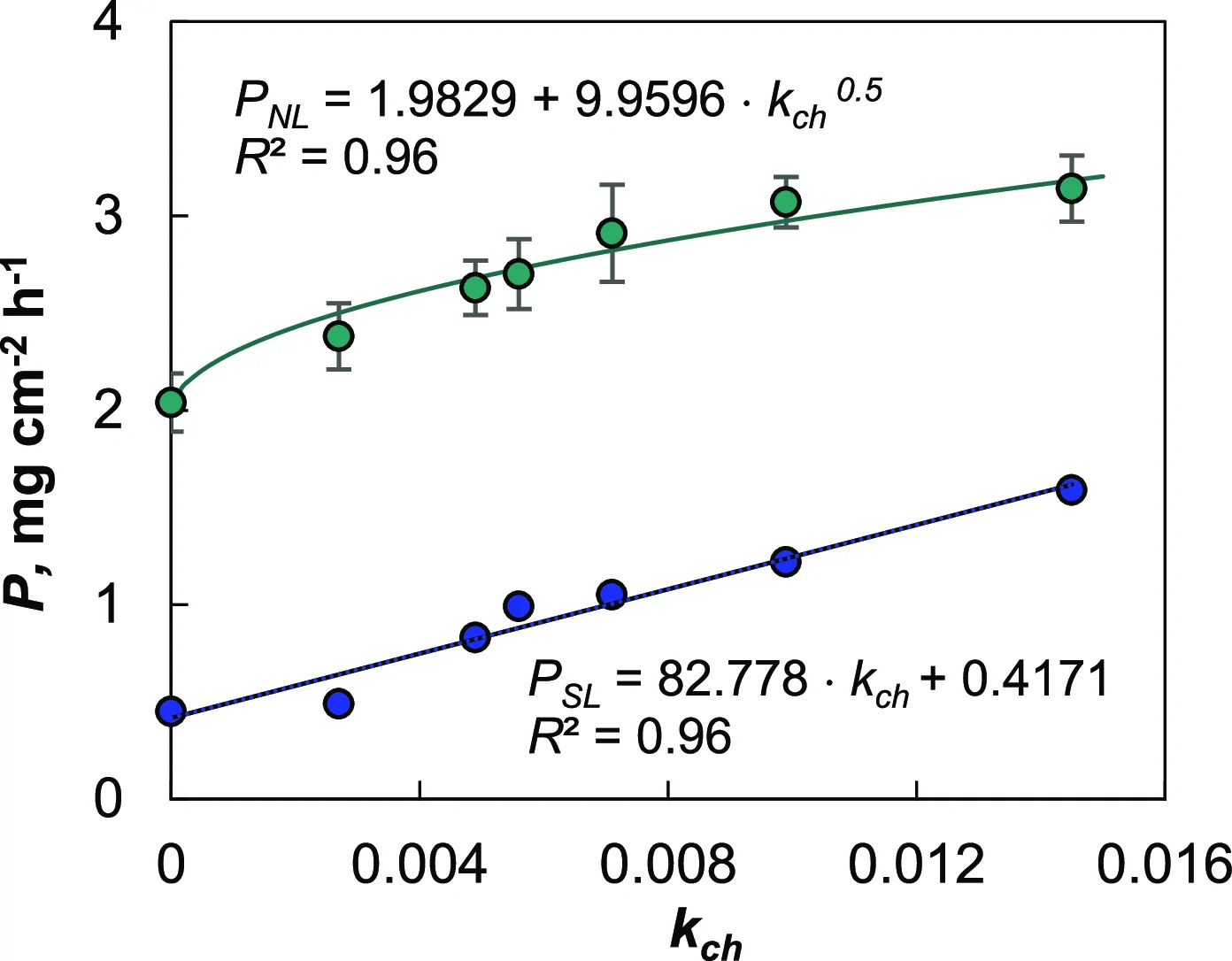
Dependence of water vapor permeability (*P*) of the top layer (NL and SL) on the transfer channels coefficient *k*_ch_ (error bars represent the standard deviation (*SD*); for SL, *SD* values ranged from 0.028 to 0.086).

For the NL layer, the regression model has a clear physical interpretation. The intercept of the regression equation (1.9829 mg cm^–2^ h^–1^) represents the predicted water vapor permeability of the unperforated material (*k*_ch_ = 0). This value is in good agreement with the experimentally measured water vapor permeability of the unperforated NL (2.04 mg cm^–2^ h^–1^). The slope of the regression indicates that water vapor permeability increases approximately linearly with the square root of the moisture transport channel coefficient, implying that the incremental improvement in permeability decreases as the channel coefficient increases.

For the SL layer, the regression model also has clear physical interpretation. The intercept of the regression equation (0.4171 mg cm^–2^ h^–1^) represents the predicted water vapor permeability of the unperforated material (*k*_ch_ = 0). This value is in good agreement with the experimentally measured water vapor permeability of the unperforated SL cm^–2^ h^–1^). The slope represents the increase in water vapor permeability with increasing moisture transport channel coefficient, reflecting the contribution of the additional open moisture transport channels created by perforation.

At the highest channel density (*k*_ch_ = 0.0145), water vapor permeability increases markedly for both materials, with a more pronounced relative effect in synthetic leather (increases by 3.5 times, from 0.45 mg cm^–2^ h^–1^ up to 1.59 mg cm^–2^ h^–1^).

The introduction of open cylindrical through-thickness moisture transport channels in the top layer (NL, SL) leads to a substantial increase in water vapor permeability (Figure 4). This enhancement arises from a modification of the dominant transport mechanism induced by the channel networks. When the channel dimensions greatly exceed the size of the penetrant molecules, resistance to molecular motion within the channels becomes negligible compared with that in the surrounding solid matrix.^22,37,38^ As a result, the open channels act as low-resistance pathways that facilitate vapor transmission. Under these conditions, water vapor transport through the channels is governed by Fickian diffusion in air, driven by the concentration gradient across the material in the absence of temperature gradients and external air flow.

Perforation of the top layer (NL, SL) alters the water vapor transport mechanism by introducing an additional Fickian diffusion pathway through open channels (Figure 5). Following the formation of these channels, the natural leather structure evolves into a dual-scale porous system comprising the intrinsic microporous collagen matrix and an engineered network of open channels. In perforated NL, water vapor transport thus proceeds via two parallel pathways: diffusion through the collagen matrix (Figure 4a) and Fickian diffusion in air through the open channels (Figure 5a)

7 $$J_{NLperf}=\left(1-k_{ch}\right)J_{NL}+k_{ch}J_{channel}$$

8 $$J_{channel}=-D_{air}\frac{dC}{dx}$$

where *k*_ch_ is the fraction of the surface area occupied by the channels, (1 *– k*_ch_) is the unperforated area fraction of the leather matrix, *J*_channel_ is the water vapor transfer flux in the cylindrical channel, and *D*_air_ is the diffusion coefficient in air (*D*_air_ ≫ *D*_eff_).

**Figure 5:**
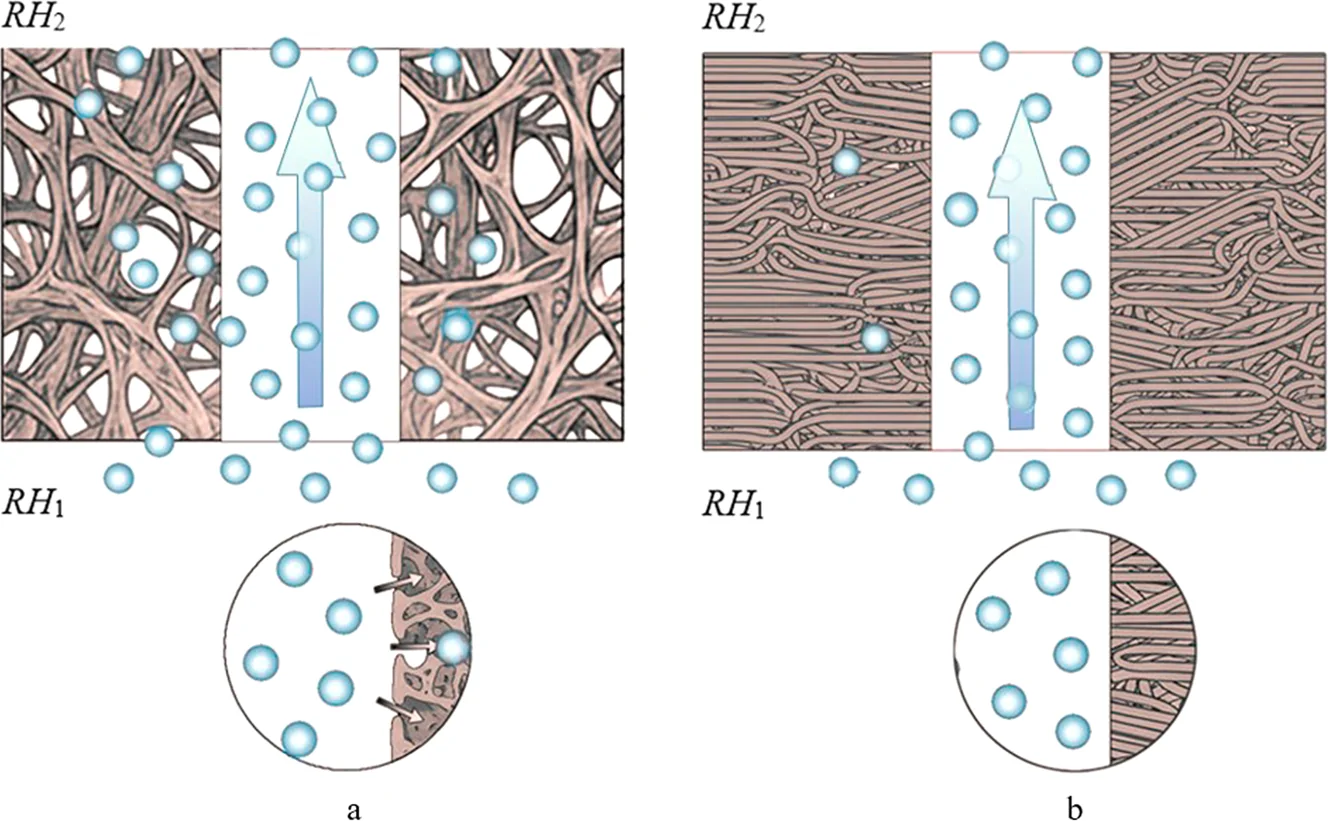
Water vapor transfer mechanisms in natural leather (a) and synthetic leather (b) after the incorporation of open moisture-transport channel networks; *RH*_1_ > *RH*_2_.

Perforation also increases the effective internal surface area of the cylindrical channels, facilitating deeper penetration of water vapor into the material structure (Figure 5a). As a result, interactions between water molecules and the polar functional groups of the hydrophilic NL matrix were enhanced. Accordingly, the dependence of water vapor permeability of the NL layer on the moisture transfer channel coefficient (Figure 4) is best described as a linear relationship with the square root of the moisture transport channel coefficient (*k*_ch_^0.5^), reflecting the evolving contribution of multiple moisture transport mechanisms.

In synthetic leather (SL), water vapor molecules dissolve into the dense, nonporous surface layer structure (Figure 3b), occupying the free volume between tightly packed polymer chains. Upon the formation of moisture transport channels, the material transitions into a dual-transport system, in which solution–diffusion through the polymer matrix (Figure 3b) is complemented by Fickian diffusion in air (Figure 5b) through the channel network

9 $$J_{SLperf}=\left(1-k_{ch}\right)J_{sol‐diff}+k_{ch}J_{channel}$$

This leads to a fundamental transformation in transport behavior, as the diffusivity in air is significantly higher *D*_air_ ≫ *D*_polymer_ than in the polymer matrix, such that even a small fraction of the SL surface converted into channels results in a substantial increase in overall vapor transport.

In contrast to natural leather (NL), synthetic leather (SL) exhibits weak interactions with water molecules because of its hydrophobic nature. Water vapor entering through the perforated surface and penetrating into the underlying nonporous structure has a negligible effect on solubility within the polymer free volume. Consequently, the water vapor permeability of SL increases linearly with the moisture transfer channel coefficient (Figure 4), reflecting the dominant contribution of Fickian diffusion in air through the open channels by the vapor concentration gradient.

The formation of a network of open channels via perforation of the top layer alters the water vapor transport mechanism by introducing an additional Fickian diffusion pathway through air, driven by the vapor concentration gradient (Figure 6).

**Figure 6:**
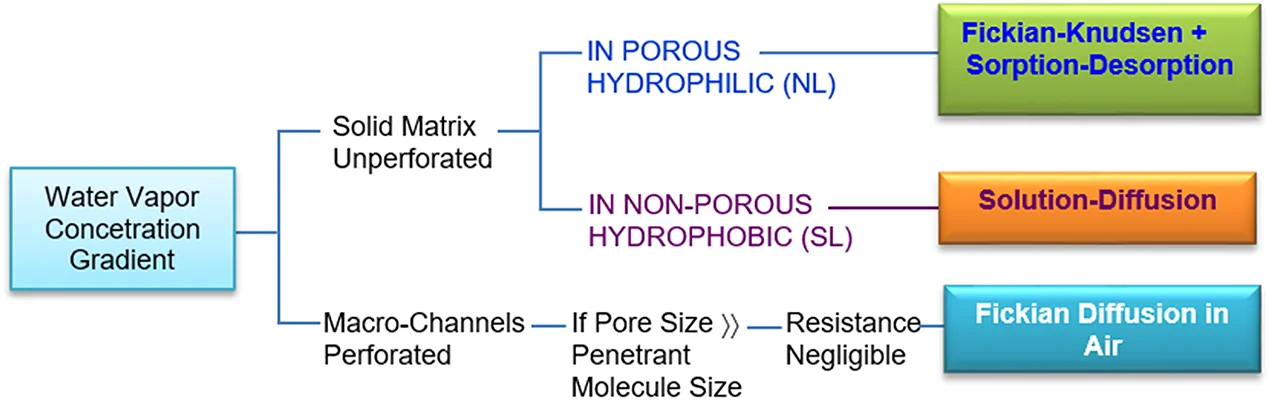
Comprehensive water vapor transfer mechanisms at a concentration gradient.

This modification leads to a significant increase in water vapor permeability and, consequently, influences the water vapor absorption behavior of the multilayer systems. Figures 7 and 8 illustrate the relationships among the moisture transfer channel coefficient, the water vapor permeability of the top layer, and the absorption capacity of the material systems. The regression equations are empirical models fitted to the experimental data, and the power-law exponents should not be interpreted as fundamental physical constants but rather as empirical parameters describing the observed behavior within the investigated range of channel coefficients.

**Figure 7:**
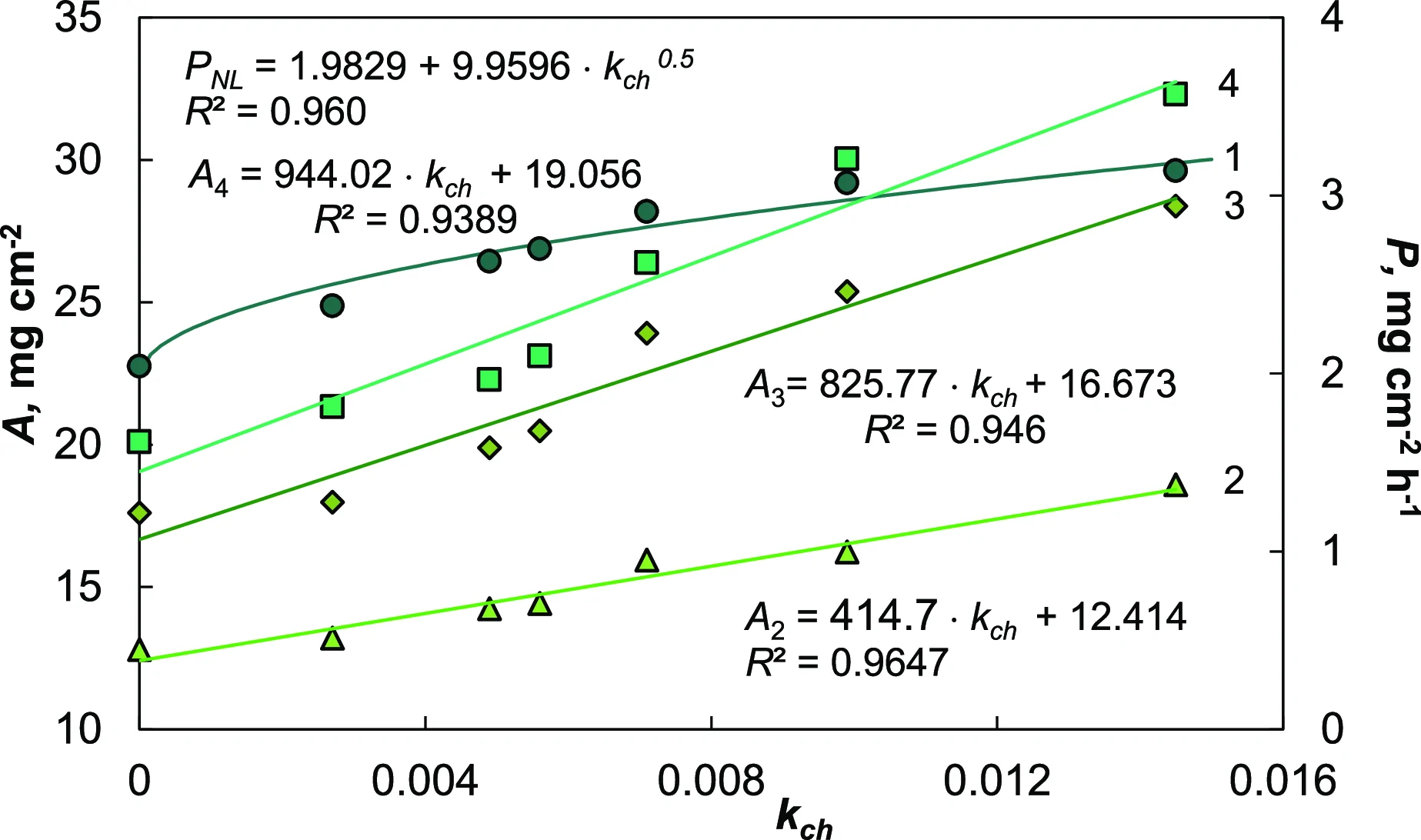
Dependence of water vapor permeability *P* (1) of the perforated top layer NL and water vapor absorption (*A*_*i*_) of the material’s systems (2 – NL + NC; 3 – NL + CF; 4 – NL + NC + CF) on the transfer channels coefficient *k*_ch_ (error bars represent the standard deviation (*SD*); *SD* values ranged from 0.39 to 0.51 for NL + NC, from 0.51 to 0.73 for NL + CF, and from 0.50 to 0.70 for NL + NC + CF).

**Figure 8:**
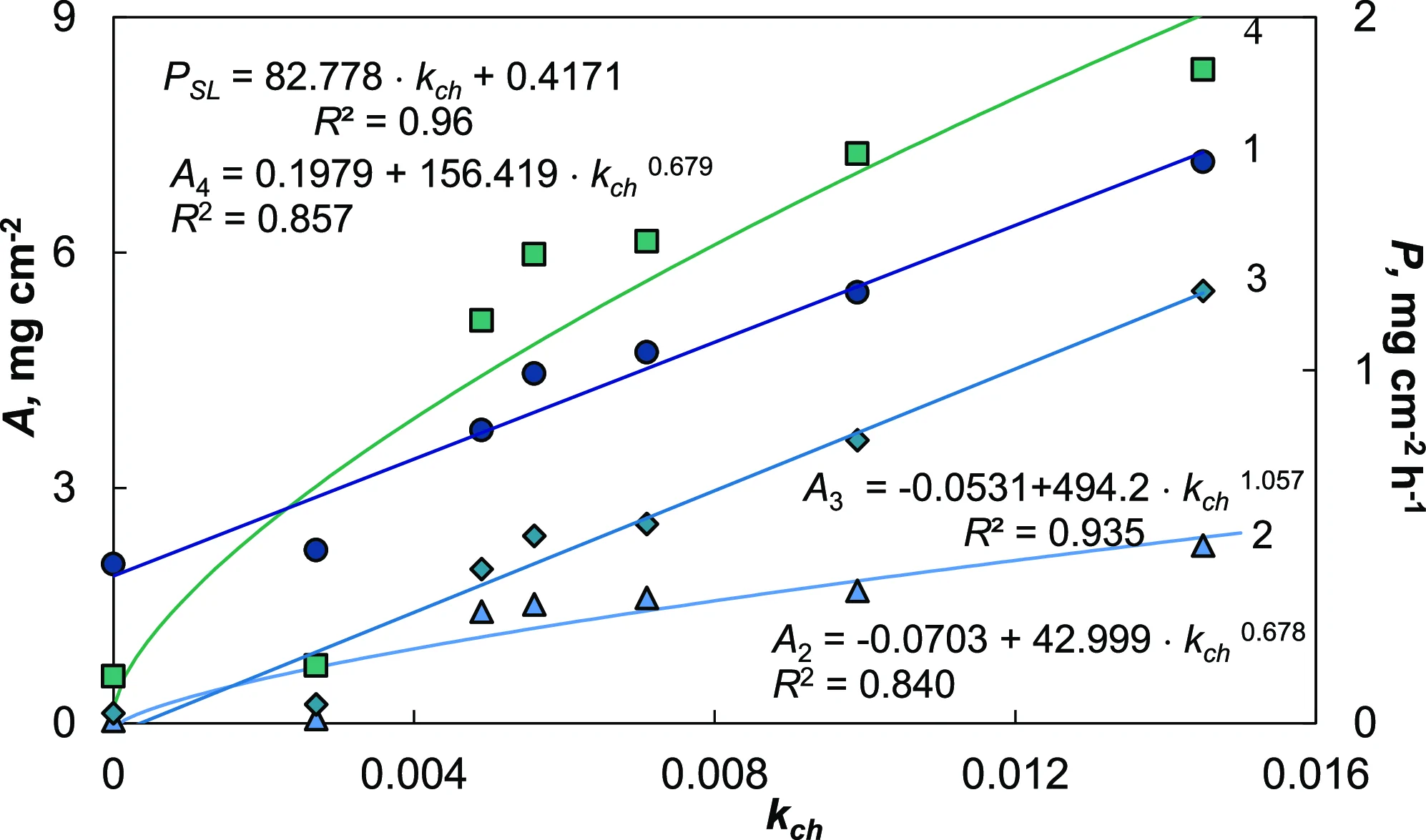
Dependence of water vapor permeability *P* (1) of the perforated top layer SL and water vapor absorption *A*_*i*_ of the material systems (2 – SL + NC; 3 – SL + CF; 4 – SL + NC + CF) on the transfer channels coefficient *k*_ch_ (error bars represent the standard deviation (*SD*); *SD* values ranged from 0.045 to 0.286 for SL + NC, from 0.051 to 0.481 for SL + CF, and from 0.043 to 0.392 for SL + NC + CF).

Increasing the perforation parameters of the top layer, and thus its water vapor permeability, leads to a corresponding increase in water vapor absorption, the extent of which depends on the material system’s composition (Figure 7). Expanding the effective channels’ surface area in natural leather enhances the water vapor absorption of the NL + NC system by approximately 45%, while increases of about 60% are observed for the NL + CF and NL + NC + CF systems relative to nonperforated counterparts. The absorption of multilayer systems is further governed by the moisture-absorbing capacity of the inner layers. Since cardboard (CF) exhibits approximately 1.2 times higher water vapor absorption than natural cork (NC), systems incorporating CF (NL + CF and NL + NC + CF) demonstrate 1.5 and 1.7 times higher moisture uptake, respectively, compared to the NL + NC system. However, the addition of a third layer provides only a marginal benefit, with the NL + NC + CF system showing just a 1.1 increase relative to the NL + CF system. The introduction of open cylindrical moisture transport channels significantly improves the effective utilization of the sorption capacity of multilayer systems. Consequently, systems with perforated natural leather achieve approximately 60% (NL + NC and NL + NC + CF) and up to 80% (NL + CF) of the total sorption capacity of their constituent materials.

The introduction of open moisture transport channels in synthetic leather (SL) leads to a substantial increase in the water vapor absorption of multilayer systems (Figure 8). Due to the negligible moisture transport properties of synthetic leather, increasing the channel area fraction and the corresponding vapor permeability significantly facilitates moisture transfer to the underlying layers of the system. As a result, the water vapor absorption of the SL + NC system increases by up to 73 times, while the SL + CF and SL + NC + CF systems exhibit increases of up to 44 and 14 times, respectively. In addition, the effective utilization of sorption capacity improves by up to 11%, 21%, and 18%, respectively, compared to systems with a nonperforated top layer (Figures 2 and 8).

Comparative results for water vapor absorption in systems with unperforated and perforated top layers (*d* = 1 mm, ρ = 1.84 cm^–2^) of natural leather (NL) and synthetic leather (SL) are presented in Figure 9.

**Figure 9:**
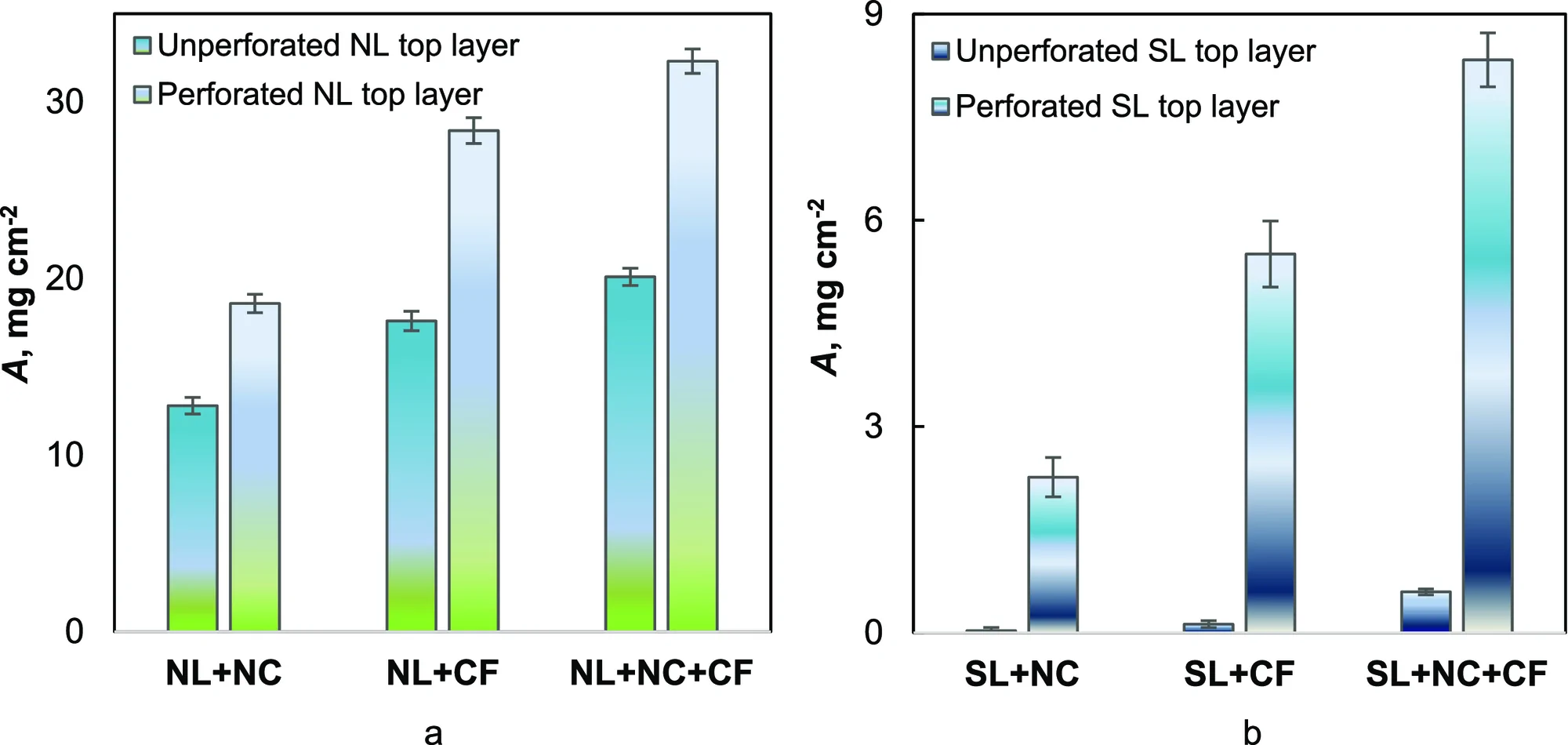
Water vapor absorption of material systems with an unperforated and perforated NL (a) and SL (b) top layer.

As shown, perforation leads to markedly different levels of enhancement depending on the top-layer material’s nature and properties. In NL-based systems, the intrinsic ability of natural leather to absorb and transmit moisture results in consistently high sorption performance. The introduction of open moisture transport channels increases vapor permeability by approximately 1.5 times, leading to a moderate increase in systems’ vapor absorption of about 45–61% compared to nonperforated systems (Figure 9a). Consequently, the water vapor absorption increased from 12.8–20.1 mg cm^–2^ for the nonperforated systems to 18.6–32.3 mg/cm^2^ for the perforated systems.

In contrast, perforation of synthetic leather produces a substantially greater relative effect. The formation of moisture transport channel networks increases the water vapor permeability of the SL layer by up to 3.5 times, resulting in a 14–73-fold increase in the water vapor absorption of the corresponding multilayer systems (Figure 9b). However, despite this substantial relative increase, the perforated SL top layer still exhibits relatively low water vapor permeability (1.59 mg cm^–2^ h^–1^). Consequently, the water vapor absorption of multilayer systems with an SL top layer increases from 0.03–0.60 mg cm^–2^ in the nonperforated systems to 2.26–8.33 mg cm^–2^ in the perforated systems. These results demonstrate that the introduction of open channels, enabling an additional Fickian diffusion pathway, is particularly effective in systems where the top layer exhibits low vapor permeability, while the underlying layers possess high sorption capacity.

Although the introduction of open moisture transport channels significantly improves water vapor transport, increasing the moisture transport channel coefficient may involve a trade-off between enhanced thermophysiological comfort and the mechanical performance of the perforated material system because of the reduction in the effective load-bearing cross-sectional area. For internal footwear insole systems, this effect may be associated primarily with compressive behavior and long-term durability. However, the evaluation of the mechanical properties was beyond the scope of the present study. Future studies should therefore investigate the relationship between enhanced moisture transport and the mechanical performance of perforated multilayer insole systems.

Thus, the overall moisture transport behavior of multilayer systems can be tailored by balancing the intrinsic properties of the constituent materials with geometrically introduced diffusion pathways, providing a basis for enhancing thermophysiological comfort while considering the potential mechanical implications of increased perforation.

## 4 Conclusions

The composition and structural configuration of two- and three-layer polymer-based systems influence thermophysiological comfort. The moisture transport behavior of multilayer systems can be tuned by balancing intrinsic material properties with geometrically engineered diffusion pathways. The introduction of open moisture transport channels modifies the governing transport mechanism by enabling an additional Fickian diffusion pathway, thereby significantly enhancing vapor transmission. Increasing the moisture transport channel coefficient (up to 0.0145) enhanced the water vapor permeability of natural leather by 1.5 times and of synthetic leather by 3.5 times. Consequently, the water vapor absorption of the corresponding multilayer systems increased by approximately 45–61% for natural leather systems (from 12.8–20.1 to 18.6–32.3 mg cm^–2^) and by 14–73 times for synthetic leather systems (from 0.03–0.60 to 2.26–8.33 mg cm^–2^). Despite the large relative increase observed for synthetic leather systems, their absolute water vapor absorption remained lower than that of natural leather systems. The channel network also improved the effective utilization of sorption capacity, increasing it to approximately 60–80% in natural leather systems and 11–21% in synthetic leather systems. Altering moisture transport mechanisms through geometrically engineered diffusion pathways establishes the principles for moisture management in multilayer polymer systems, particularly in clothing and accessory applications.

## Supplementary Material



## Notes

Mean values and corresponding standard deviations of the experimental results (PDF)
