# Supplementary material for: Altering Moisture Transport Mechanisms in Multilayer Material Systems via Open Channel Networks for Enhanced Comfort
Source: ACS Omega. 2026 Oct 12;11(42):65176–84. doi: 10.1021/acsomega.6c07464 (PMC13717790; doi:10.1021/acsomega.6c07464)
Supplement: Supplementary file 1 [file ao6c07464_si_001.pdf]

Altering Moisture Transport Mechanisms in Multilayer Materials Systems via Open Channel Networks for Enhanced Comfort

Ada Gulbinienė*[,1], Vaida Dobilaitė [2], Milda Jucienė [2]

[1] Faculty of Mechanical Engineering and Design, Kaunas University of Technology,

Studentu Street 56, Kaunas LT- 44029, Lithuania

[2] Institute of Architecture and Construction, Kaunas University of Technology, Tunelio Street 60,

Kaunas LT- 44029, Lithuania

*Email: ada.gulbiniene@ktu.lt.

Table S1. Mean values and standard deviations of experimental measurements.

Figure	Materials	Mean values, A, mg cm^{-2}	Standard deviation	
Figure 2.	1 – SL	0.03	0.031	
	4 – SL+NC	0.03	0.048	
	5 – SL+CF	0.13	0.05	
	6 – L+NC+CF	0.60	0.048	
**Figure**	**Material**	k_{ch}	**Mean values, P_{SL}, mg cm^{-2}h^{-1}**	**Standard deviation**
Figure 3, 8	SL	0	0.45	0.028
		0.0027	0.49	0.029
		0.0049	0.83	0.050
		0.0056	0.99	0.057
		0.0071	1.05	0.045
		0.0099	1.22	0.073
		0.0145	1.59	0.086
**Figure**	**Material**	k_{ch}	**Mean values, P_{NL}, mg cm^{-2}h^{-1}**	**Standard deviation**
Figure 3, 7	NL	0	2.04	0.15
		0.0027	2.38	0.17
		0.0049	2.63	0.14
		0.0056	2.7	0.18
		0.0071	2.91	0.25
		0.0099	3.07	0.13
		0.0145	3.14	0.17
**Figure**	**Materials**	k_{ch}	**Mean values, A, mg cm^{-2}**	**Standard deviation**
Figure 7	2 – NL+NC	0	12.80	0.51
		0.0027	13.21	0.48
		0.0049	14.24	0.51
		0.0056	14.42	0.44
		0.0071	15.95	0.39
		0.0099	16.23	0.47
		0.0145	18.58	0.50

**Figure**	**Materials**	k_{ch}	**Mean values, A, mg cm^{-2}**	**Standard deviation**
Figure 7	3 – NL+CF	0	17.60	0.68
		0.0027	17.98	0.57
		0.0049	19.89	0.51
		0.0056	20.49	0.59
		0.0071	23.91	0.73
		0.0099	25.38	0.68
		0.0145	28.38	0.73
**Figure**	**Materials**	k_{ch}	**Mean values, A, mg cm^{-2}**	**Standard deviation**
Figure 7	4– NL+NC+CF	0	20.10	0.70
		0.0027	21.34	0.65
		0.0049	22.29	0.59
		0.0056	23.12	0.50
		0.0071	26.39	0.61
		0.0099	30.04	0.59
		0.0145	32.31	0.69
**Figure**	**Materials**	k_{ch}	**Mean values,** A, mg cm^{-2}	Standard deviation
Figure 8	2 – SL+NC	0	0.031	0.045
		0.0027	0.052	0.089
		0.0049	1.421	0.150
		0.0056	1.514	0.147
		0.0071	1.598	0.178
		0.0099	1.685	0.233
		0.0145	2.263	0.286
**Figure**	**Materials**	k_{ch}	**Mean values, A, mg cm^{-2}**	**Standard deviation**
Figure 8	3 – SL+CF	0	0.125	0.051
		0.0027	0.237	0.179
		0.0049	1.963	0.259
		0.0056	2.388	0.383
		0.0071	2.537	0.353
		0.0099	3.605	0.361

		0.0145	5,509	0.481

**Figure**	**Materials**	k_{ch}	**Mean values, *A*, mg cm^{-2}**	**Standard deviation**
Figure 8	4 – SL+NC+CF	0	0.595	0.043
		0.0027	0.734	0.151
		0.0049	5.132	0.268
		0.0056	5.977	0.259
		0.0071	6.141	0.350
		0.0099	7.257	0.354
		0.0145	8.333	0.392
